# Prognostic implications of ΔNp73/TAp73 expression ratio in core-binding factor acute myeloid leukemia

**DOI:** 10.1038/s41408-024-01086-8

**Published:** 2024-06-24

**Authors:** Maria L. Salustiano-Bandeira, Amanda Moreira-Aguiar, Diego A. Pereira-Martins, Juan L. Coelho-Silva, Isabel Weinhäuser, Pedro L. França-Neto, Aleide S. Lima, Ana S. Lima, Anemari R. Baccarin, Fernanda B. Silva, Manuela A. de Melo, Fernanda S. Niemann, Luciana Nardinelli, César A. Ortiz Rojas, Bruno K. Duarte, Aderson S. Araujo, Elisa A. Azevedo, Clarice N. Morais, Lorena L. Figueiredo-Pontes, Jan J. Schuringa, Gerwin Huls, Israel Bendit, Eduardo M. Rego, Sara T. Olalla Saad, Fabiola Traina, Marcos A. Bezerra, Antonio R. Lucena-Araujo

**Affiliations:** 1 Department of Genetics, Federal University of Pernambuco, Recife, Brazil; 2 Department of Hematology, Cancer Research Centre Groningen, University Medical Centre Groningen, University of Groningen, Groningen, The Netherlands; 3 Department of Medical Imaging, Hematology, and Oncology, Medical School of Ribeirao Preto, University of São Paulo, Ribeirao Preto, Brazil; 4 Hematology and Transfusion Medicine Center, University of Campinas, Campinas, Brazil; 5 Hematology Division, Faculty of Medicine, University of Sao Paulo, Sao Paulo, Brazil; 6 Department of Internal Medicine, University of Campinas, Campinas, Brazil; 7 Department of Internal Medicine, Hematology and Hemotherapy Foundation of Pernambuco, Recife, Brazil; 8 Department of Virology, Fundação Oswaldo Cruz, Instituto de Pesquisas Aggeu Magalhães, Recife, Brazil

**Keywords:** Health sciences, Medical research, Translational research

Dear Editor,

The *TP73* gene is a member of the *TP53* family and, due to differential promoter usage, it may be alternatively transcribed into the transcriptionally active full-length (*TAp73*) or inactive NH2-terminal truncated (*ΔNp73*) isoforms. While the encoded full-length TAp73 largely mimics p53 activities in experimental systems [1], the transactivation-deficient ΔNp73 isoform exerts a dominant-negative effect on p53 and TAp73 functions [2]. Given that both isoforms can occur simultaneously in the cell, the oncogenic potential of the *TP73* gene in cancer is dictated by the balance between ΔNp73 and TAp73 isoforms. In the clinical context, we have previously demonstrated that a high *ΔNp73*/*TAp73* expression ratio is independently associated with inferior outcomes in patients with acute promyelocytic leukemia [3]. Additionally, we have recently demonstrated that patients exhibiting overexpression of ΔNp73 can be grouped into a particular subset of acute myeloid leukemia (AML) with a molecular signature and clinical outcomes very similar to *TP53*-mutant AML [4], reinforcing the prognostic relevance of the *TP73* gene and its isoforms in human cancers. Now, we are interested in determining whether the *ΔNp73*/*TAp73* ratio has clinical implications in core-binding factor (CBF)-AML, another well-defined subtype of AML which, although categorized as favorable-risk AML [5], has a substantial portion of patients who die from relapsed disease after intensive chemotherapy [6].

Between February 2004 and May 2022, 136 cytogenetically and/or molecularly confirmed cases of de novo CBF-AML (median age: 47 years, range: 18–79 years; 51% male) were enrolled. Details about cohort characterization can be found in the Supplemental data. The majority of the patients (114 patients, 84%) were younger than 60 years old and received intensive cytotoxic chemotherapy. Induction therapy included daunorubicin (60 or 90 mg/m^2^ daily for 3 days) and cytarabine (100 or 200 mg/m^2^ daily for 7 days), followed by two or three cycles of consolidation therapy with high doses cytarabine (1.5 g/m^2^ or 3 g/m^2^ for 3 days). None of the patients underwent autologous or allogeneic bone marrow transplantation in first remission. Complete remission was assessed by bone marrow examination 28 days after each course of chemotherapy. The remaining 22 patients aged 60 years were treated with low-dose of cytarabine in combination with etoposide, thioguanine, and idarubicin, or received palliative care. The study adhered to the tenets of the Declaration of Helsinki, and informed consents were obtained from all patients or their relatives. The study was approved by the Internal Review Board (CAAE #47769821.7.0000.5208).

The transcript levels of *TP73* isoforms (*ΔNp73* and *TAp73*) were quantified using SYBR Green Dye method (Promega) (Supplemental data). Only pretreatment bone marrow mononuclear cells were analyzed. Strategies for cohort dichotomization are described in the Supplemental data. All calculations were performed using Stata statistical data analysis software version 14.1 (StataCorp, College Station, TX, USA), statistical package for the social sciences 19.0, and R 3.3.2 (The CRAN project, www.r-project.org) software. *P*-values were two-sided with a significance level of 0.05. Details for statistical analysis and clinical endpoints were published elsewhere [3].

Initially, we conducted an exploratory analysis to determine whether the isolated expression of *TAp73* or *ΔNp73* had clinical implications in CBF-AML. As indicated in Supplemental Table 2, *TAp73* alone had no association with achieving complete remission (CR), overall survival (OS), and disease-free survival (DFS) rates. On the other hand, patients with high *ΔNp73* expression had lower 5-years OS rate (19%, 95% CI: 10–30) than patients with low *ΔNp73* expression (35%, 95% CI: 22–48) (*P* = 0.024; Supplemental Fig. 2). Univariate analysis showed that *ΔNp73* was associated with poor OS (hazard ratio, HR: 1.59, 95% confidence interval, CI: 1.1–2.41), although this result has not been consistent with the multivariable proportional hazards analysis (HR: 1.52, 95% CI: 0.99–2.31, *P* = 0.06). ΔNp73 had no association CR rate and DFS (Supplemental Table 2).

Next, we focused on the impact of *ΔNp73*/*TAp73* expression ratio in clinical outcomes. Overall, 90/136 (66%) patients achieved CR. Although a high *ΔNp73*/*TAp73* ratio was associated with lower CR rate in univariate logistic analysis (odds ratio, OR: 2.54; 95% confidence interval, CI: 1.14–5.63; *P* = 0.021), this difference was no longer significant after adjustment for sex, age and leukocyte counts (OR: 2.15; 95% CI: 0.93–4.95; *P* = 0.071). Out of the 46 patients (34%) who failed to achieve CR, 31 (23%) experienced early mortality (i.e., death within 30 days after diagnosis), which was not impacted by the *ΔNp73*/*TAp73* ratio (OR: 0.95, 95% CI: 0.39–2.29; *P* = 0.91).

**Fig. 1:**
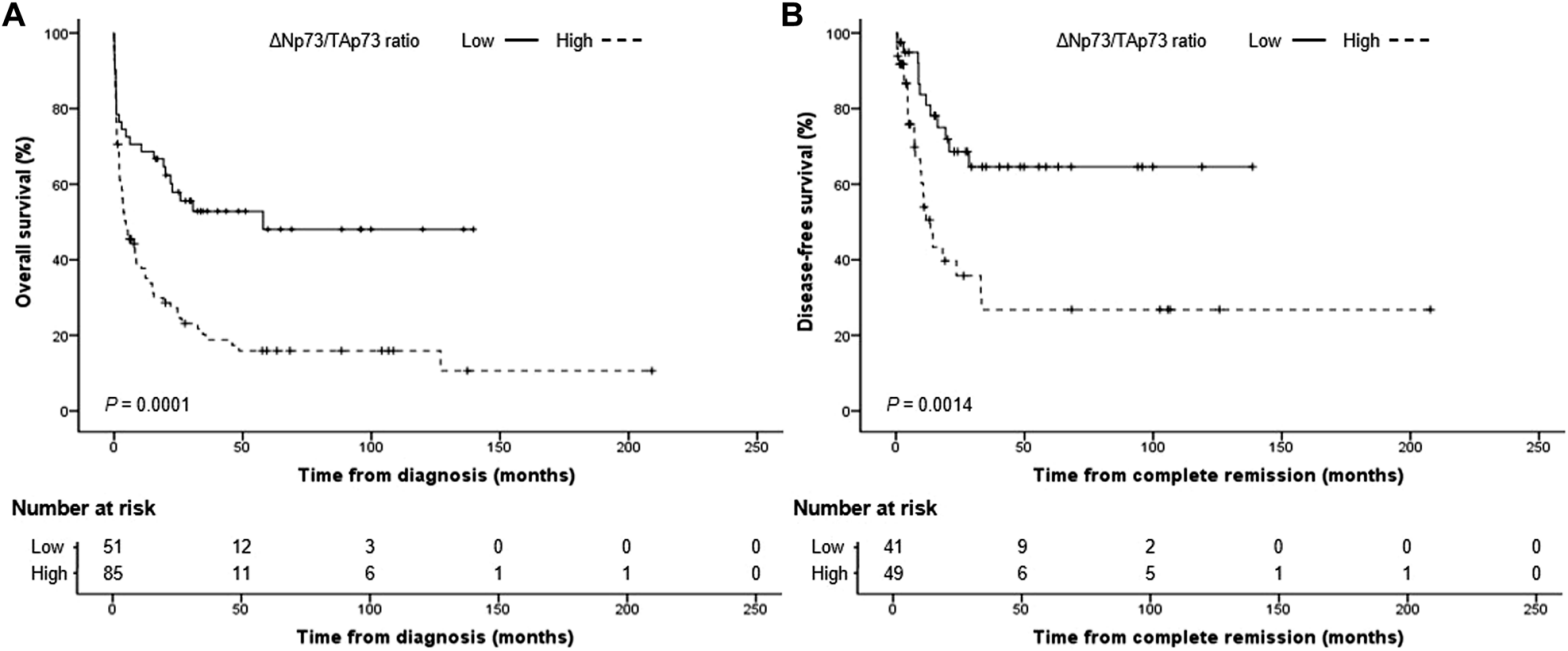
Survival results for patients with CBF-AML according to the *ΔNp73*/*TAp73* ratio. Probability of overall survival (**A**) and disease-free survival (**B**). Survival curves were estimated using the Kaplan–Meier method, and the log‐rank test was used for comparison.

With a median follow-up of 8.1 months (range: 0.1–209 months), the estimated 5-year OS was 28% (95% CI: 20–36%). Patients with a high *ΔNp73*/*TAp73* ratio had a significantly lower 5-year OS (16%, 95% CI: 9–25%) than patients with a low *ΔNp73*/*TAp73* ratio (48%, 95% CI: 32–63%) (*P* = 0.0001) (Fig. 1A). In univariable analysis, we noticed that patients with a high ΔNp73/TAp73 ratio had an almost threefold higher risk of presenting poor OS (hazard ratio, HR: 2.93, 95% CI: 1.52–3.88; *P* < 0.001). Multivariable Cox proportional hazards model showed that high *ΔNp73*/*TAp73* ratio was independently associated with poor OS using sex, age, and leukocyte counts as confounders (HR: 2.22, 95% CI: 1.36-3.6; *P* = 0.001) (Supplemental Table 3). Of the 90 patients who achieved CR, 36 patients (40%) relapsed. Patients with a high *ΔNp73*/*TAp73* ratio had significantly lower 5-year DFS (27%, 95% CI: 12–44%) compared to patients with a low ratio (65%, 95% CI: 46–78%) (*P* = 0.0014) (Fig. 1B). High *ΔNp73*/*TAp73* ratio was associated with poor DFS in both univariable (HR: 2.98, 95% CI: 1.48–6; *P* = 0.002) and multivariable analyses (HR: 3.1, 95% CI: 1.48–6.36; *P* = 0.003) (Supplemental Table 3).

Frequently classified as a favorable-risk disease [5], increasing reports have described CBF-AML as a heterogeneous condition, in which up to 40% of patients die of treatment failure [7]. Some studies argue that such clinical heterogeneity is caused by secondary genetic abnormalities [7, 8] which, in turn, could result in substantial differences between patients with t(8;21) and inv(16) [9]. Hence, the current literature supports the need for enhanced genomic characterization in CBF-AML to identify distinct prognostic groups of patients. Other evidence suggests this difference may also be observed when comparing cohorts from different clinical contexts. For instance, we have noticed that the clinical outcomes of our patients were significantly inferior to those reported by Begna et al. [10]. In their controlled study of 70 patients with CBF-AML, the authors reported a near 100% CR rate in patients uniformly treated with “7 + 3” protocols, with 61% of the patients (27 at risk) remaining alive five years post-diagnosis. The authors also showed that pre-emptive allogeneic hematopoietic stem cell transplant might reduce the risk of relapse. In agreement, Takahashi et al. [11] reported similar results when investigating regimens’ response and treatment-related mortality based on alternative protocols in elderly patients with CBF-AML. Although we did not intend to compare clinical data between cohorts, our data are in sharp contrast with the both studies (in particular, CR and OS rates). However, we should consider some points of concern. First, our study was conducted within a public healthcare system in a low- and middle-income country, which means many variables cannot be fully controlled. These include delays in initiating treatment, lack of adequate infrastructure for stem cell transplantation, and drug unavailability with subsequent adaptations in treatment protocols [12]. Additionally, our setting faces some challenges related to infection control, particularly regarding invasive fungal infections, which accounts for substantial morbidity during treatment [12, 13]. Together, the clinical heterogeneity of CBF-AML appears to be influenced not only by disease biology but also by patient demographics and healthcare infrastructure. This reinforces the importance of conducting studies in diverse economic and clinical contexts and highlights the need for treatment strategies adjusted to local conditions and patient-specific factors.

Here, we provided the first evidence that a higher *ΔNp73/TAp73* ratio is associated with poor outcomes in patients with CBF-AML intensively treated with standard protocols. Although we have not explored the biological significance of *ΔNp73*/*TAp73* ratio in CBF-AML at a functional level, our results suggest that the TP53 pathway may be indirectly compromised. It remains to be explored whether the imbalance between *ΔNp73* and *TAp73* isoforms may be caused by differential stability of each transcript or due to a particular mechanism that dictate which isoform will be preferentially transcribed in a tumor cell. In this context, our group recently described a new poor prognostic subgroup of *TP53*-wild type patients that behaves similarly to *TP53*-mutant AML. Patients included in this new group frequently present increased expression of *ΔNp73*. Using molecular profiling of 823 AML patients, we noticed that the transcriptional and metabolic program of ΔNp73-AML patients share strong similarities with *TP53*-mutant patients [4]. This resemblance includes enrichment for stemness signatures, co-occurrence with poor prognosis mutations, and increased cell proliferation and resistance against several standard-of-care cytotoxic therapies in AML. Because *TP53* is rarely mutated in de novo AML [14], these data further highlight the relevance of the *ΔNp73*/*TAp73* ratio in refining patient risk-stratification and predicting chemotherapy response in AML, even in diseases with such a favorable prognosis, such as CBF-AML.

### Supplementary information

Supplemental data
