# Supplementary material for: Prognostic implications of ΔNp73/TAp73 expression ratio in core-binding factor acute myeloid leukemia
Source: Blood Cancer J. 2024 Jun 24;14(1):102. doi: 10.1038/s41408-024-01086-8 (PMC11196665; doi:10.1038/s41408-024-01086-8)
Supplement: Supplementary file 1 — Supplemental data [file 41408_2024_1086_MOESM1_ESM.docx]

**MYELOID NEOPLASIA**

**Prognostic implications of ΔNp73/TAp73 expression ratio in core-binding factor acute myeloid leukemia**

Maria L Salustiano-Bandeira,^1^ Amanda Moreira-Aguiar,^1^ Diego A Pereira-Martins,^1,2^ Juan L Coelho-Silva,^1^ Isabel Weinhäuser,^2^ Pedro L Franca-Neto,^1^ Aleide S Lima,^1^ Ana S Lima,^3^ Anemari R Baccarin,^3^ Fernanda B Silva,^3^ Manuela A de Melo,^3^ Fernanda S Niemann,^6^ Luciana Nardinelli,^4^ Cesar Ortiz,^4^ Bruno K Lino,^5,6^ Aderson S Araujo,^7^ Elisa A Azevedo,^8^ Clarice N Morais,^8^ Lorena L Figueiredo-Pontes,^3^ Jan J Schuringa,^2^ Gerwin Huls,^2^ Israel Bendit,^4^ Eduardo M Rego,^4^ Sara Saad,^5,6^ Fabiola Traina,^3^ Marcos A Bezerra,^1^Antonio R Lucena-Araujo^1^*

**Affiliations**: ^1^ Department of Genetics, Federal University of Pernambuco, Recife, Brazil; ^2^ Department of Hematology, Cancer Research Centre Groningen, University Medical Centre Groningen, University of Groningen, Groningen, the Netherlands; ^3^ Department of Medical Imaging, Hematology, and Oncology, Medical School of Ribeirao Preto, University of São Paulo, Ribeirao Preto, Brazil; ^4^ Hematology Division, LIM31, Faculdade de Medicina, University of Sao Paulo, Sao Paulo, Brazil; ^5^ Department of Internal Medicine, University of Campinas, Campinas, Brazil; ^6^ Hematology and Transfusion Medicine Center, University of Campinas, Campinas, Brazil; ^7^ Department of Internal Medicine, Hematology and Hemotherapy Foundation of Pernambuco, Recife, Brazil; ^6^ Department of Virology, Fundação Oswaldo Cruz, Centro de Pesquisas Aggeu Magalhães, Recife, Brazil.

*** Corresponding author:** Antonio R Lucena-Araujo. Department of Genetics, Federal University of Pernambuco. Av. Prof. Moraes Rego, 1235, Recife, PE 50670-901, Brazil. Tel: +55-81-2126-7825. Fax: +55-81-2126-7825.

E-mail: [antonio.araujo@ufpe.br](mailto:antonio.araujo@ufpe.br)

*Supplemental data*

**METHODS**

*Patients and study design*

We performed a retrospective cohort study investigating the relative expression of ΔNp73 and TAp73 isoforms in patients with core-binding factor acute myeloid leukemia (CBF-AML) at diagnosis. Only patients diagnosed with *de novo* CBF-AML were included. Between February 2004 and May 2022, 136 patients were diagnosed with CBF-AML at four Brazilian reference centers specialized on acute myeloid leukemia (AML) treatment. All patients were required to have a bone marrow samples at diagnosis. AML diagnosis was determined following WHO criteria. The presence of t(8;21)(q22;q22) and inv(16)(p13q22) or t(16;16)(p13;q22) [hereafter named as t(8;21) and inv(16)] was assessed by conventional karyotyping, while the corresponding rearrangements (*RUNX1-RUNX1T1* and *CBFβ-MYH11*) were confirmed by reverse transcription-polymerase chain reaction. All materials used for cytogenetic analyses or molecular characterization were obtained at diagnosis and were processed in the reference laboratories of each participating center. Collaborative data collection from each center occurred after confirming diagnosis.

Overall, 80 patients had t(8;21), while 56 patients had inv(16). Except for a higher platelet counts in patients with inv(16), the clinical outcomes were similar between patients with t(8;21) and inv(16) (Supplemental data). Given the similarities between patients and the limited number of samples in each group, we combined both cytogenetic subgroups for subsequent analyses. An overview of patient characteristics can be found in the Supplemental table 1. All patients or their relatives gave their written informed consent for scientific evaluations. The study was approved by the Internal Review Board (CAAE #47769821.7.0000.5208) and adhered to the tenets of the Declaration of Helsinki.

*Cohort dichotomization*

To dichotomize patients according to *ΔNp73*/*TAp73* ratio, we opted to optimize the cut off selection using the cutpointr package using R software (1). Subsequently, we used survival receiver operating characteristic curve analysis and the C-index to validate this dichotomization strategy using overall survival as a primary endpoint. Following these criteria, patients with *ΔNp73*/*TAp73* ratio lower than 0.92 were assigned to the low expression group (51 patients; 37%), while those with *ΔNp73*/*TAp73* ratio equal or higher than 0.92 were assigned to the high expression group (85 patients; 63%). For comparison purposes, we also dichotomized patients according to the primary data from *TAp73* (low *TAp73* expression < 0.41; 58 patients, 43%) and *ΔNp73* (low *ΔNp73* expression < 2.25; 71 patients, 52%).

*Gene expression assays*

Real-time quantitative polymerase chain reaction (RT-qPCR) assays were performed using patient-derived cDNA. All samples were tested in duplicates on MicroAmp optical 96-well plates using a QuantStudio®5 Real-Time PCR System (Thermo Fisher Scientific). The transcript levels of *ΔNp73* and *TAp73* were quantified by RT-qPCR using the SYBR Green Dye method (Promega), following the manufacturer’s instructions. Briefly, PCR reaction was performed in a 10-µl reaction mixture containing 5µl of 2x Go-Taq qPCR MasterMix, 200 ng cDNA, and 0.6µl of each primer at 10nM. The amplification conditions were as follows: 50°C for 2 minutes and 95°C for 10 minutes, followed by 40 cycles of 95°C for 15 seconds and 64°C for 1 minute. Melting curves for each PCR product were then generated.

The comparative Cq (cycle of quantification) method was used to determine the relative expression levels of *ΔNp73* and *TAp73*, with the *ACTB* and the *HPRT1* genes as endogenous controls. The difference in Cq number (ΔCq = Cq_ΔNp73 or TAp73_ – Cq_endogenous_) was calculated for each replicate. The gene expression of each *TP73* isoform was calculated relative to a reference cDNA known to express both isoforms (2) (THP-1, a human acute myeloid leukemia cell line). The expression values of *ΔNp73* and *TAp73* were then calculated as a relative quantification (RQ) = 2^-∆∆Cq^, with ∆∆Cq being = ΔCq_patients_ – ΔCq_THP-1 cell line_. The results are presented as a ratio between the two isoforms, hereinafter called *ΔNp73/TAp73* ratio*.* Primer sequences are summarized in Supplemental table 2.

**RESULTS**

The main aim of the present study was to conduct an exploratory analysis of clinical outcomes of patients with CBF-AML related to the balance between the *TP73* isoforms (ΔNp73 and TAp73). However, we also explored potential differences between patients with t(8;21) and inv(16) based on clinical heterogeneity frequently reported between these two conditions (3–9). As summarized in the Supplemental table 1, clinical and baseline characteristics were similar between patients, except for a higher platelet counts in patients with inv(16) (*P*=0.021). Supplemental figure 1 shows the overall survival curve of patients according to the t(8;31) and inv(16).

| Supplemental table 1. Clinical and baseline characteristics of patients with CBF-AML. | | | | | | | | |
| --- | --- | --- | --- | --- | --- | --- | --- | --- |
| Characteristic | | All patients | | CBF-AML | | | | *P* value |
|  |  |  |  | t(8;21) | | inv(16) | |  |
|  |  | No. | % | No. | % | No. | % |  |
| Age, years | |  |  |  |  |  |  | 0.211 |
|  | 18-40 years | 54 | 39.7 | 36 | 45 | 18 | 32.1 |  |
|  | 40-60 years | 60 | 44.1 | 34 | 42.5 | 26 | 46.4 |  |
|  | 60 years and older | 22 | 16.2 | 10 | 12.5 | 12 | 21.4 |  |
| Median (range) | | 47.5 (18, 79) | | 45 (18, 77) | | 48.5 (18, 79) | | 0.06 |
| Sex | |  |  |  |  |  |  | 0.605 |
|  | Female | 67 | 49.3 | 41 | 51.3 | 26 | 46.4 |  |
|  | Male | 69 | 50.7 | 39 | 48.8 | 30 | 53.6 |  |
| Leukocyte counts, ×10^9^/L, median (range) | | 18.1 (0.6, 24.3) | | 15.1 (0.6, 21.1) | | 27.1 (0.7, 24.3) | | 0.091 |
| Hemoglobin, g/dL, median (range) | | 7.8 (3.3, 13.4) | | 7.6 (3.3, 13.4) | | 8.2 (4, 11.1) | | 0.411 |
| Platelet counts, ×10^9^/L, median (range) | | 23 (1, 151) | | 20 (1, 136) | | 33 (5, 151) | | 0.023* |
| CR, No. (%) | | 90 (66.2) | | 55 (68.7) | | 35 (62.5) | | 0.467 |
| 3-y OS, % (95% CI) | | 28 (19, 63) | | 30 (21, 42) | | 23 (11, 35) | | 0.376 |
| 3-y DFS, % (95% CI) | | 46 (33, 58) | | 53 (37, 67) | | 30 (11, 51) | | 0.221 |
| NOTE: * Indicates statistically significant differences.  Abbreviations: CR, complete remission; OS, overall survival; DFS, disease-free survival; WBC, white blood cells. | | | | | | | | |

| **A.** | 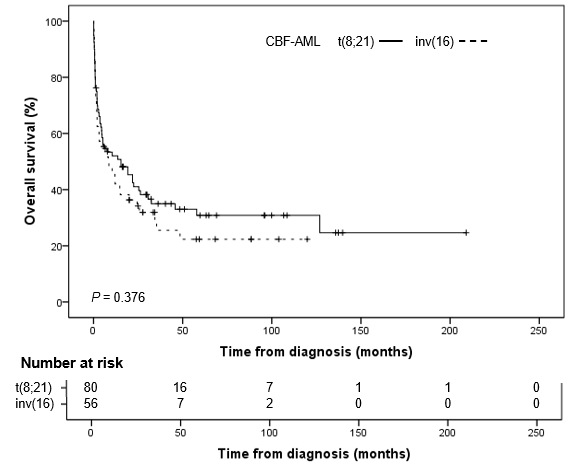 |
| --- | --- |
| **Supplemental figure 1**. The probability of overall survival in patients with CBF-AML. Survival curves were estimated using the Kaplan–Meier method, and the log‐rank test was used for comparison. | |

Next, we performed an exploratory analysis to ascertain if the individual expression of TAp73 or ΔNp73 had clinical significance in CBF-AML. Supplemental table 2 summarize the outcomes of patients according to the ΔNp73/TAp73 ratio, and according to the isolated expression of TAp73 and ΔNp73 expression. Supplemental figure 2 shows the probability of overall survival in patients with CBF-AML according to the (A) TAp73 and (B) ΔNp73 expression.

Supplemental table 3 shows the multivariable Cox proportional hazards analysis for overall survival and disease-free survival for CBF-AML patients.

| Supplemental table 2. Summary of outcomes of patients according to the *ΔNp73*/*TAp73* ratio, *TAp73* and *ΔNp73* expression. | | | | | | |  |  |
| --- | --- | --- | --- | --- | --- | --- | --- | --- |
| All patients, No. (%): 136 (100) | CR | OS | | DFS | | | |  |
|  | % | No. | 5-yr % (95% CI) | | No. | 5-yr % (95% CI) | | |
| Low *ΔNp73*/*TAp73* ratio | 78 | 51 | 48 (32, 63) | | 41 | 65 (46, 78) | | |
| High *ΔNp73*/*TAp73* ratio | 59 | 85 | 16 (9, 25) | | 49 | 27 (12, 44) | | |
| *P*-value | 0.025* |  | 0.0001* | |  | 0.0014* | | |
| All patients, No. (%): 136 (100) | CR % | OS | | DFS | | | |  |
|  |  | No. | 5-yr % (95% CI) | | No. | 5-yr % (95% CI) | | |
| Low *TAp73* expression | 64 | 48 | 28 (16, 43) | | 29 | 47(27, 64) | | |
| High *TAp73* expression | 67 | 88 | 26 (16, 37) | | 60 | 46 (29, 61) | | |
| *P*-value | 0.714 |  | 0.382 | |  | 0.384 | | |
| All patients, No. (%): 136 (100) | CR % | OS | | DFS | | | |  |
|  |  | No. | 5-yr % (95% CI) | | No. | 5-yr % (95% CI) | | |
| Low *ΔNp73* expression | 67 | 31 | 35 (22, 48) | | 18 | 49 (32, 65) | | |
| High *ΔNp73* expression | 66 | 105 | 19 (10, 30) | | 71 | 42 (23, 60) | | |
| *P*-value | 0.996 |  | 0.024* | |  | 0.087 | | |
| Abbreviations: CR: complete remission; OS: overall survival; DFS: disease-free survival; CI: confidence interval.  * Indicates differences that are statistically significant (*P* < 0.05). | | | | | | |  |  |

| 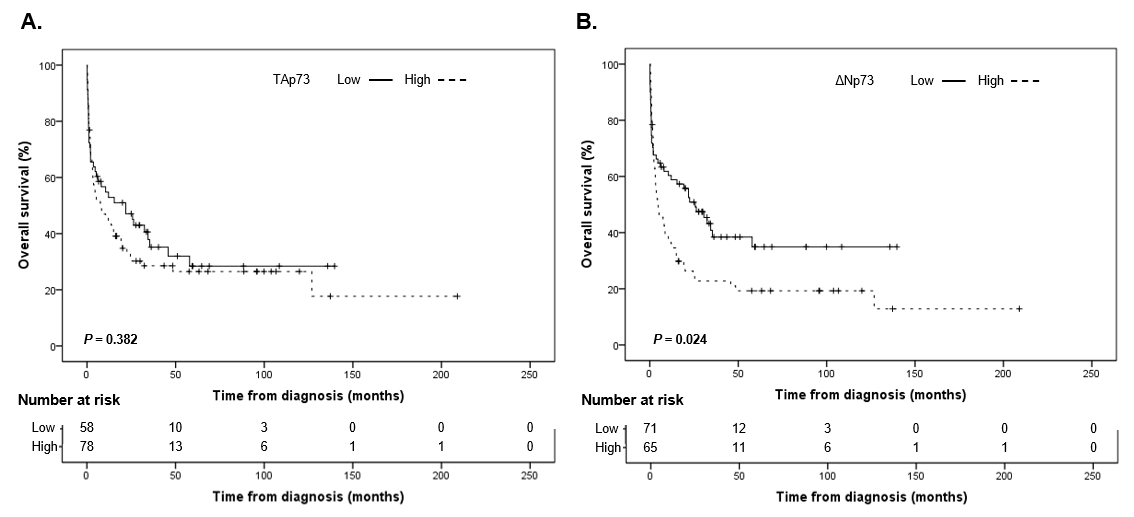 |
| --- |
| **Supplemental figure 2**. The probability of overall survival in patients with CBF-AML according to the (A) TAp73 and (B) ΔNp73 expression. Survival curves were estimated using the Kaplan–Meier method, and the log‐rank test was used for comparison. |

| Supplemental Table 3. Multivariable Cox model for overall survival and disease-free survival. | | | | | | | | | | |
| --- | --- | --- | --- | --- | --- | --- | --- | --- | --- | --- |
| Variable | Overall survival | | | | Disease-free survival | | | | |  |
|  | HR | 95% CI | | *P* value | HR | 95% CI | | *P* value |  |  |
| *ΔNp73*/*TAp73* ratio: high *versus* low | 2.22 | 1.36 | 3.6 | 0.001 | 3.1 | 1.48 | 6.36 | 0.003 |  |  |
| Sex: male *versus* female | 0.87 | 0.58 | 1.32 | 0.538 | 0.97 | 0.49 | 1.93 | 0.945 |  |  |
| Age (years): continuous variable | 1.6 | 0.95 | 2.7 | 0.072 | 1.22 | 0.44 | 3.37 | 0.697 |  |  |
| Leukocyte counts (× 10^9^/L): continuous variable | 1.24 | 0.79 | 1.95 | 0.336 | 1.1 | 0.51 | 2.26 | 0.84 |  |  |
| Abbreviations: HR: hazard ratio; CI: confidence interval. | | | | | | | | |  |  |

**REFERENCES**

1. Thiele C, Hirschfeld G. **cutpointr** : Improved Estimation and Validation of Optimal Cutpoints in *R*. J Stat Softw. 2021;98(11).

2. Pluta A, Nyman U, Joseph B, Robak T, Zhivotovsky B, Smolewski P. The role of p73 in hematological malignancies [Internet]. Vol. 20, Leukemia. Nature Publishing Group; 2006 [cited 2020 Oct 29]. p. 757–66. Available from: www.nature.com/leu

3. Paschka P, Du J, Schlenk RF, Gaidzik VI, Bullinger L, Corbacioglu A, et al. Secondary genetic lesions in acute myeloid leukemia with inv(16) or t(16;16): a study of the German-Austrian AML Study Group (AMLSG). Blood. 2013 Jan 3;121(1):170–7.

4. Ishikawa Y, Kawashima N, Atsuta Y, Sugiura I, Sawa M, Dobashi N, et al. Prospective evaluation of prognostic impact of KIT mutations on acute myeloid leukemia with RUNX1-RUNX1T1 and CBFB-MYH11. Blood Adv. 2020 Jan 14;4(1):66–75.

5. Jourdan E, Boissel N, Chevret S, Delabesse E, Renneville A, Cornillet P, et al. Prospective evaluation of gene mutations and minimal residual disease in patients with core binding factor acute myeloid leukemia. Blood. 2013 Mar 21;121(12):2213–23.

6. Solh M, Yohe S, Weisdorf D, Ustun C. Core‐binding factor acute myeloid leukemia: Heterogeneity, monitoring, and therapy. Am J Hematol. 2014 Dec 27;89(12):1121–31.

7. Bullinger L, Rücker FG, Kurz S, Du J, Scholl C, Sander S, et al. Gene-expression profiling identifies distinct subclasses of core binding factor acute myeloid leukemia. Blood. 2007 Aug 15;110(4):1291–300.

8. Mosna F, Papayannidis C, Martinelli G, Di Bona E, Bonalumi A, Tecchio C, et al. Complex karyotype, older age, and reduced first‐line dose intensity determine poor survival in core binding factor acute myeloid leukemia patients with long‐term follow‐up. Am J Hematol. 2015 Jun;90(6):515–23.

9. Qin W, Chen X, Shen HJ, Wang Z, Cai X, Jiang N, et al. Comprehensive mutation profile in acute myeloid leukemia patients with RUNX1- RUNX1T1 or CBFB-MYH11 fusions. Turkish Journal of Hematology. 2022 Apr 21;
